# Therapeutic Antibodies in Medicine

**DOI:** 10.3390/molecules28186438

**Published:** 2023-09-05

**Authors:** Prerna Sharma, Rahul V. Joshi, Robert Pritchard, Kevin Xu, Maya A. Eicher

**Affiliations:** Geisinger Commonwealth School of Medicine, Scranton, PA 18509, USA

**Keywords:** therapeutic antibodies, medicine, drug discovery, drug development, protein engineering

## Abstract

Antibody engineering has developed into a wide-reaching field, impacting a multitude of industries, most notably healthcare and diagnostics. The seminal work on developing the first monoclonal antibody four decades ago has witnessed exponential growth in the last 10–15 years, where regulators have approved monoclonal antibodies as therapeutics and for several diagnostic applications, including the remarkable attention it garnered during the pandemic. In recent years, antibodies have become the fastest-growing class of biological drugs approved for the treatment of a wide range of diseases, from cancer to autoimmune conditions. This review discusses the field of therapeutic antibodies as it stands today. It summarizes and outlines the clinical relevance and application of therapeutic antibodies in treating a landscape of diseases in different disciplines of medicine. It discusses the nomenclature, various approaches to antibody therapies, and the evolution of antibody therapeutics. It also discusses the risk profile and adverse immune reactions associated with the antibodies and sheds light on future applications and perspectives in antibody drug discovery.

## 1. Introduction

Antibodies, or immunoglobulins, are the effector molecules of the humoral immune response. They are antigen-neutralizing molecules, produced by plasma B-cells in our body, that protect us by destroying pathogens and preventing their propagation. The main functions performed by antibodies are neutralization, opsonization, activation of the complement pathway, and antibody-dependent cell-mediated cytotoxicity. With the advancement of contemporary molecular science, their natural potential has been harnessed to develop therapeutics to treat diseases ranging from cancer to autoimmune diseases.

Antibody engineering is regarded as a rich seam, and its scope is quite evident, as seen by the great variety of engineered antibodies on the market. As a means of natural defense, our immune systems produce antibodies to help us circumvent infections and diseases. Today, therapeutic antibodies are the most well-characterized and potent weapons used by physicians to fight many diseases. Protein engineering has been extensively applied to modify the properties and functions of antibodies [1]. The landmark work in developing the first monoclonal antibodies [2] and producing the first FDA-approved antibody, muromonab-CD3 (Orthoclone, OKT3, developed for acute transplant rejection) [3], paved the way for more than 100 therapeutic and diagnostic antibodies.

The ‘big 5’ that drive the therapeutic antibody industry are infliximab, adalimumab, trastuzumab, bevacizumab, and rituximab. Infliximab and adalimumab are specific for tumor necrosis factor (TNF) and are used for treating Crohn’s disease, rheumatoid arthritis, and plaque psoriasis. Trastuzumab and bevacizumab are specific to human epidermal growth factor receptor 2 (HER2) and vascular endothelial growth factor A (VEGFA), respectively, and are used for the treatment of different cancers. Lastly, rituximab is a CD20-specific antibody used for both rheumatoid arthritis and non-Hodgkin’s lymphoma. Antibodies generate revenue in the millions, accounting for the highest-earning category in biological drugs. The main indications for monoclonal antibodies are cancer and autoimmune diseases, collectively making up ~78% of the total antibody sales in the US [4].

### 1.1. Antibody Structure and Its Types

**Figure 1:**
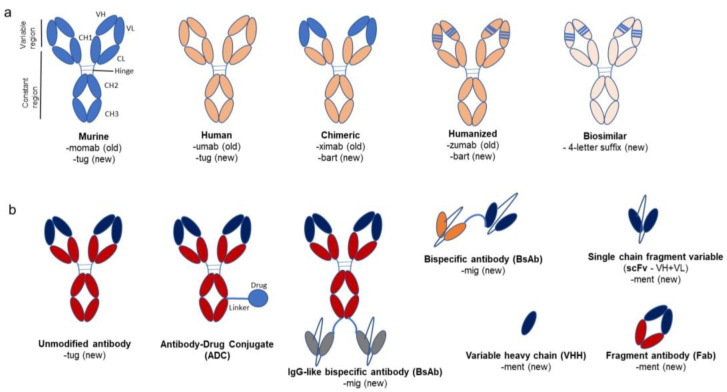
Overview of monoclonal antibodies and their variants with old and new naming conventions. (**a**) shows murine, human, chimeric, and humanized antibodies. The chimeric antibody has a variable region of murine origin (light blue) and a constant region sourced from a human antibody (coral orange). The humanized antibody is mostly human except for the complementarity-determining regions (CDRs, light blue lines) or hypervariable regions, which are of murine origin. The old and new nomenclature for antibodies are mentioned beneath each picture. The murine and human antibodies are unaltered, so they contain the stem “tug” compared to the chimeric and humanized antibodies, which carry the stem “bart”. The biosimilars have a random 4-letter suffix. (**b**) shows the variants of antibodies available, viz. antibody-drug conjugate (ADC), bispecific antibodies, fragment antibodies (with variable (VL + VH) and constant domain 1 (CH1 and CL) from both heavy and light chains), and single-chain fragment antibodies like single-chain fragment variable (scFv − VH + VL) and variable heavy domain (VHH). This figure is based on a paper by Wang et al. (2022) [17].

A fully assembled and functional antibody (with a molecular weight of ~150 kDa) consists of four polypeptide chains, i.e., two identical light and two identical heavy chains (Figure 1). The sizes of the light and heavy chains are 25 and 50 kDa, respectively. The two chains are joined to each other by covalent disulfide linkages, along with some non-covalent interactions like hydrophobic, ionic, salt bridging, and hydrogen bonding interactions that contribute to the assembly of the structure. The light and heavy chains further comprise two (a variable, VL and a constant, CL) and four immunoglobulin (a variable, VH and three constants, CH1, CH2, and CH3) domains, respectively. The variable domains comprise the amino terminals of both chains. The amino terminal varies and changes in specificity according to the antigen. These variable domains (VH and VL) assemble to form the antigen binding site. In the variable domain, the region that exhibits hypervariability is called the complementarity determining region (CDR), and the region in the variable domain that remains largely the same or unchanged is called the framework region (FW). There are three CDRs each in the heavy and light chains. They are present in connecting loops projecting out of the β strands, arranged in two sheets to form a β sandwich with Greek-key topology. The HCDR3 (heavy-chain complementarity determining region 3) is the most variable. The region beyond the variable domain is called the constant region, comprising the immunoglobulin domains (one, CL and three, CH1, CH2, and CH3). The antibody comprises six immunoglobulin domains (two in the light chain and four in the heavy chain), namely, variable light (VL), constant light (CL), variable heavy (VH), and constant heavy (CH1, CH2, and CH3). The general structure of this domain consists of a sandwich of two β sheets composed of antiparallel β strands connected to each other with a disulfide bond.

There are various versions of antibodies, ranging from completely unaltered antibodies either from mice (murine) or humans to altered versions like chimeric, humanized, and fragmented antibodies produced via protein engineering approaches (Figure 1). Murine monoclonal antibodies, derived from mice and rats of the Muridae family, are IgGs that kicked off the modern development of therapeutic antibodies. They have a similar structure to human antibodies, with two heavy chains and two light chains forming a Y-shaped molecule [5]. Despite similarities, there are differences in the sequence and glycosylation of the constant domains, which lead to an immunogenic response. The antibody also contains distinct CDR-H3 regions in the variable heavy domain, contributing to differences in amino acid composition and immune response [6]. Chimeric antibodies often contain human constant domains and murine variable domains [7]. Humanized antibodies are largely human antibodies with murine hypervariable regions that underwent targeted mutations grafted onto the variable domains [8,9].

Camelid antibodies, derived from camels, llamas, and alpacas, are notably composed of only a heavy-chain variable domain (VHH), lacking the CH1 chain found in human antibodies [10]. Owing to their small size, these are often termed nanobodies, and they are an example of the fragmented antibodies pursued by antibody designers [11]. Like the variable new antigen receptor (VNAR) [12] found in sharks, the single VHH domain was found to exhibit the same affinity as an entire human antibody [13]. The high affinity of VHH for a large repertoire of sequences may be due to a long CDR3 loop with hypervariable regions slightly different from those in mice and humans [14]. It has been thought that the paratopes of camelid antibodies can uniquely bind epitopes on concave surfaces, which is distinct from the binding of human antibodies [15]. This may be attributed to the presence of a non-canonical disulfide bond in addition to the conserved or canonical disulfide bond. The non-canonical disulfide bond between CDR1 and CDR3 is a distinguishing feature of VHHs and is hypothesized to reduce the entropy associated with the immobilization of the CDR3 loop upon antigen binding [16].

Examples of antibody fragments are the single-chain fragment variable (scFv) that comprises a variable heavy and variable light domain (VH + VL) connected by a linker; the fragment antibody (Fab), which has a variable part and a constant heavy domain 1 from both light and heavy chains (VH + CH1 and VL + CL); or the single domain variable heavy domain (VHH) from camelids. These antibodies can also be modified by linking them to radionuclides, enzymes, drugs, or toxins or by adding elements (bispecific antibodies or CAR-T) to them to help improve their effector responses. The next section provides more details about these modification methods.

### 1.2. Types of Therapy with Antibodies

**Figure 2:**
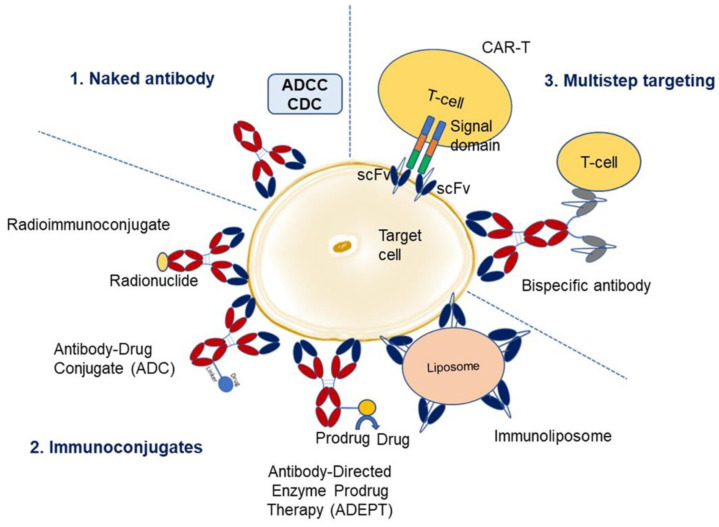
Overview of monoclonal antibody therapies. There are three main categories. The first category utilizes naked antibody and kills the target cell by antibody-dependent cell-mediated cytotoxicity (ADCC) and complement-dependent cytotoxicity (CDC). The second category comprises immunoconjugates where an antibody is linked to a radionuclide (radioimmunoconjugate), drug (antibody-drug conjugate), enzyme (antibody-directed enzyme prodrug therapy, ADEPT), or liposome (immunoliposome). The third category utilizes a multistep targeting approach where the antibodies are engineered to be specific either to the chimeric T-cell signaling domain/receptor (CAR-T) or T-cell marker (bispecific antibodies), along with specificity towards target cell for enhanced effector function (adapted from Carter 2001 [18], and Lu et al. 2020 [19]).

Antibodies have been used in different ways to target the diseases listed in Figure 2 (adapted from Carter 2001 [18], and Lu et al. 2020 [19]). These fall into three main categories viz., naked antibody, immunoconjugate, and multistep targeting. Naked antibodies kill the target cell by antibody-dependent cell-mediated cytotoxicity (ADCC) or complement-dependent cytotoxicity (CDC). Immunoconjugates have antibodies tagged with a radionuclide, drug, toxin, cytokine, or enzyme. In radioimmunotherapy, radioactively labeled murine antibodies specific to cellular antigens are utilized to target lymphomas and radio-sensitive malignancies. Murine antibodies are chosen based on their high immunogenicity and rapid tumor clearance, thereby limiting radiation exposure. I-131-Tositumomab is one such example and has been used in the treatment of non-Hodgkin’s lymphoma [20]. Another immunoconjugate therapy is antibody-directed enzyme prodrug therapy (ADEPT), which utilizes cancer antibodies linked to drug-activating enzymes to target malignant cells. A nontoxic prodrug, a substrate for the enzyme, is administered and is cleaved by the enzyme to release an inactivating component from the prodrug, generating a potent cytotoxic agent or molecule that can kill cancer cells. There has been limited success in clinical trials of ADEPT [21]. Antibodies are directly linked to drugs to generate antibody-drug conjugates (ADCs), which have the potential to kill the target cells (e.g., cancerous cells), sparing normal cells. So far, twelve ADCs have received market approval (all for oncotherapies), and several investigational ADCs are currently in pre-clinical and clinical trials. Additionally, antibodies are conjugated to liposomes (forming immunoliposomes), which carry the drug or therapeutic nucleotides through the body. Immunoliposomes are effectively used in vivo to direct tumor-suppressing genes into tumors, utilizing an antibody fragment targeted to the human transferrin receptor. Gene delivery through immunoliposomes has been accomplished in brain and breast cancer tissue [22]. The third category, bispecific antibodies, consists of antibodies engineered to have specificity for two ligands or receptors, therefore enhancing their therapeutic effect. Bispecific antibodies help mediate antibody-engaged effector functions [23]. Finally, CAR-T involves the insertion of a gene for a chimeric T-cell receptor antibody that targets specific markers on T-cells in a way such that the engineered cells target and kill the cancer cell [24]. CAR-T antibodies have received a great deal of attention recently, owing to their clinical benefits and success with cancer patients [25,26].

### 1.3. Evolution of Antibody Therapeutics

Production of therapeutic antibody drugs has evolved from the use of murine antibodies, first created via hybridoma technology, to the latest humanized or human versions using transgenic human-mice platforms [17]. The first monoclonal antibodies developed in mouse models [2,3] were found to be immunogenic due to their foreign origin and produced a strong human anti-mouse antibody (HAMA) response in patients [27], which was observed as neurotoxicity (post-HAMA response) in patients who received muromonab-CD3 [28]. To circumvent these problems, antibodies were designed as chimeric variants, which were structures like human antibodies that also retained binding properties due to the use of murine variable regions. The first United States (US) Federal Drug Administration (FDA)-approved chimeric antibody, Abciximab (an anti-GPIIb/IIIa Fab, approved in 1994), was targeted towards platelet aggregation [29] and displayed a resolved HAMA response. However, it needed further optimization, as the variable region was of murine origin and could elicit immunogenicity. This was followed by efforts to produce a humanized antibody, where the complementary-determining regions (CDRs) were grafted onto the human antibody scaffold [30,31]. Daclizumab (an anti-IL2 receptor antibody for transplant rejection) and bevacizumab (an anti-vascular endothelial growth factor (VEGF) antibody for metastatic colorectal cancer) are the two known humanized antibodies approved by the FDA in 1997 and 2004, respectively [32]. In 1990, Sir Gregory P. Winter utilized phage display technology to produce fully human mAbs (suffix-umab) [33]. Adalimumab (Humira), an anti-tumor necrosis factor α (TNFα) antibody, is a completely human mAb approved by the FDA in 2002 for the treatment of cancer and autoimmunity [34,35,36,37,38]. Finally, efforts were directed toward producing fully human antibodies using humanized transgenic mice platforms [39]. Examples include panitumumab and nivolumab, which target the epidermal factor receptor (EGFR) antibody [40] and the programmed cell death protein-1 (PD1), respectively [41,42].

### 1.4. Nomenclature of Antibodies

The names of antibodies provide information about the intended target, source (host), modifications, and conjugation to other molecules. The WHO published guidelines from the International Nonproprietary Name (INN) in 2014, 2017, and 2021 describing the classification of naming mAbs [43,44,45]. The nomenclature consists of a prefix, a substem (initially proposed as two and later reduced to one), and a stem (suffix). The prefix is random, as it provides a unique identity to the antibody. The substem indicates the target (e.g., “ci” for cardiovascular, “ba” for bacterial, “os” for bone, “ta” for tumor, etc.) and host (e.g., “o” for murine, “xi” for chimeric, “zu” for humanized, and “nu” for fully human). The second part of the substem, which revealed information about the host, has been eliminated after 2017, applicable only to the antibodies produced after 2017. Until 2021, the stem “mab” was used for all the antibodies and their fragments. However, after the revisions in 2021, the stem was divided into four groups. Group 1 uses the stem “tug” for full-length **u**nmodified immuno**g**lobulins, like IgG, IgA, IgM, IgE, etc., that might occur normally in the immune system. Group 2 uses “bart” for full-length anti**b**odies **art**ificial, which contain engineered constant domains with point mutations for any reason like glycation or altered complement binding. Group 3 uses “mig” for **m**ulti-**i**mmuno**g**lobulins (including bi- and multi-specific monoclonal antibodies), regardless of the format type (full length or fragments). Finally, group 4 uses the stem “ment” for monospecific antibody frag**ment**s that lack an Fc region. Biosimilar mAbs have a core name with the reference drug followed by a meaningless and unique four-letter suffix separated by a hyphen. For example, adalimumab biosimilars have distinguishing suffixes like -atto, -adbm, -adaz, -bwwd, and -afzb [46].

### 1.5. Databases for Therapeutic Antibodies

**Figure 3:**
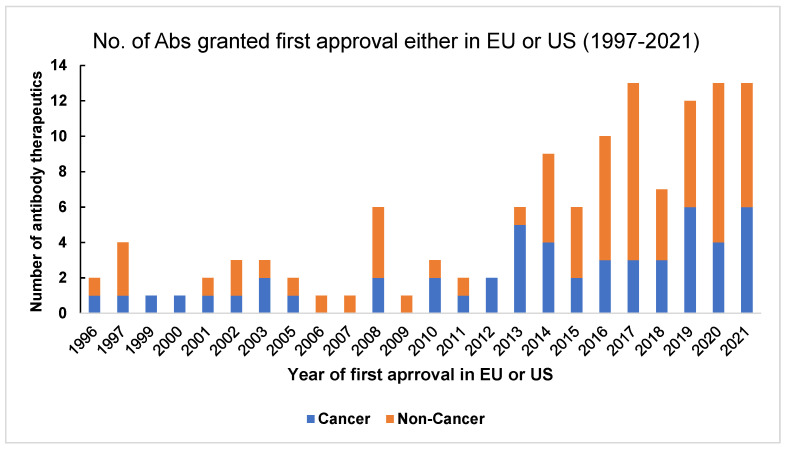
Number of antibody therapeutics in cancer and non-cancer categories that were granted first approval either in the United States (US) or the European Union (EU) from 1997–2021. This graph is adapted from The Antibody Society. Therapeutic monoclonal antibodies approved or under review in the EU or US. [47] www.antibodysociety.org/resources/approved-antibodies, accessed on 30 June 2023.

There are various databases of therapeutic antibodies that are either approved or under regulatory review. The antibody society can be accessed to receive a comprehensive review of therapeutic antibodies in the European Union (EU) and the United States (US) [47]. Figure 3 (adapted from the antibody society website, www.antibodysociety.org/resources/approved-antibodies, accessed on 30 June 2023) shows the number of antibody therapeutics in cancer and non-cancer areas that have been granted first approval either in the US or the EU. The Oxford Protein Informatics Group has helpful resources for people studying therapeutic antibodies [48]. The Therapeutic Structural Antibody database (Thera-SAbDab) is a database from OPIG [49] that provides information on immunotherapeutic variable domain sequences and their corresponding structural representatives in the database. It helps match the exact and closely related sequences [50]. Tabs is a different database for companies developing therapeutic antibodies ([51]. The Chinese antibody society, in collaboration with the antibody society, has a tracker (https://chineseantibody.org/covid-19-track/, accessed on 30 June 2023) [52] that records antibody-based therapeutics against COVID-19. This was an effort to support information in fighting the pandemic globally [52]. Information on US-FDA-approved antibodies targeted at different diseases can be found on the US-FDA website [53].

## 2. Antibodies in Different Branches of Medicine

The following sections describe the system-wise utilization or application of antibody therapeutics in treating diseases in different branches of medicine, ranging from hematology, oncology, and autoimmune disease to different organ system-related disorders like vascular, renal, neurological, etc. 

### 2.1. Antibodies in Hematological Diseases

The therapeutic use of monoclonal antibodies is prevalent in hematology conditions, particularly in hematology/oncology. Rituximab represented a breakthrough in cancer treatment as one of the first monoclonal antibodies directed against the CD20 antigen on B lymphocytes [54]. Though first approved for the treatment of relapsed indolent non-Hodgkin’s lymphoma, it has now become a standard component of treatment for follicular lymphoma (FL), diffuse large B-cell lymphoma (DLBCL), and chronic lymphocytic leukemia (CLL) [55]. Since its development, it has spurred the synthesis of additional monoclonal antibody therapies targeting CD20, which now account for over 30% of all current therapeutic monoclonal antibodies for cancer [56]. Each of these anti-CD20 antibodies functions by depleting B cells, albeit through different mechanisms, including direct cell death, antibody-dependent cellular cytotoxicity, phagocytosis, and complement-dependent cytotoxicity [57].

Emicizumab is a bispecific antibody that has emerged as a successful therapy for hemophilia A, and it works by bridging factors IXa and Xa in the coagulation cascade in order to restore the recruitment function of the deficient factor VIII [54,58]. Classic treatment for hemophilia A requires constant prophylactic infusions of factor VIII—at least two infusions a week—contributing to reduced treatment compliance [59]. However, subcutaneous administrations of emicizumab once weekly, or once every 2–4 weeks, have not only shown effectiveness in preventing bleeds but are also well tolerated [60]. 

Crizanlizumab was approved by the FDA in 2019 for use in preventing vaso-occlusive crises (VOCs) in patients with sickle cell disease [54,61]. Repetitive VOCs in these patients lead to chronic organ damage, making this a major cause of morbidity and mortality [62,63]. The pathophysiology of these crises entails the attachment of sickle cells to the vascular endothelium [61,64], mediated by the adhesion molecule P-selectin, which is also what crizanlizumab inhibits. Although it does not seem to reduce hemolysis, crizanlizumab has a significant impact on the frequency of VOCs with a low rate of adverse effects [64].

Although treatments like rituximab are effective, not all patients are able to take advantage of these treatment options due to excessive costs [65]. Biosimilars are biologic medicines with high similarity to existing biologics that offer a potential solution to this ongoing issue of hematologic monoclonal antibodies [54]. The goal in designing biosimilars is to preserve the safety, efficacy, purity, and mechanism of action of existing biologics [66] while avoiding the complexity of synthesizing large molecules like antibodies. This poses the question of whether new antibodies are true biosimilars [54]. Thus, the current goal of future developments for monoclonal antibody therapies in hematology is to make current therapies more accessible to patients while also improving and developing new treatment options.

### 2.2. Antibodies for Autoimmune Diseases

Autoimmune diseases are characterized by aberrant immune-cell function, resulting in the destruction of cells and tissues. Though these are often difficult to treat due to a personalized dysfunction of the body’s inherent immune response, a general understanding of immune cellular processes offers insight into the many possible targets for therapy, particularly cytokines and cell signaling receptors [67]. 

Rheumatoid arthritis (RA) is characterized by chronic inflammation and synovitis, leading to flares of polyarthritis and joint pain. Though the exact mechanism is unknown, pro-inflammatory mediators such as IL-6, IL-1, and TNF-α play a significant role in the synovitis and bone erosion seen in RA. Thus, these cytokines are common targets for treatment with monoclonal antibodies [68]. The first line of treatment for RA is a disease-modifying anti-rheumatic drug (DMARD), such as methotrexate. However, traditional DMARD-resistant RA can be treated using biological DMARDs such as monoclonal antibodies. Combined administration of biologics with traditional DMARDs has shown significant efficacy in randomized control trials [69]. Systemic symptoms of RA can be attributed to IL-6 overactivity, making this cytokine a key target in drug therapy. In RA, aberrant proliferation of macrophage-like synoviocytes (type-A) and fibroblast-like synoviocytes (type-B) leads to uninhibited cytokine release, especially IL-6 and TNF-α [70]. Tocilizumab, a humanized monoclonal antibody (IgG1 κ) against the membrane-bound and soluble IL-6 receptors, is especially useful in minimizing systemic RA symptoms such as fatigue, anemia, and acute phase reactions [71]. Sarilumab is another humanized monoclonal antibody (IgG1) that binds to and inhibits downstream signaling of the IL-6 receptor [72]. Sarilumab has a similar effectiveness and safety profile to tocilizumab when used for the treatment of methotrexate-resistant RA [73]. The TNF-α inhibitors infliximab and adalimumab are also approved for the treatment of RA; however, other biological DMARDs such as tocilizumab seem to be more effective in the treatment of disease symptoms and drug adherence for patients [74,75]. 

Systemic lupus erythematous (SLE) is an autoimmune disease with widely varying severity and involvement of organ systems. Due to its complex manifestations, treatment of SLE is highly individualized and depends on the specific organ involvement and the magnitude of severity. It is recommended that the majority of SLE patients be treated with hydroxychloroquine, and additional medications like monoclonal antibodies are added to their regimen as needed [76]. Belimumab, a monoclonal antibody (Ig-G1 ɣ) against B lymphocyte stimulator (BLyS), is approved for the treatment of SLE and is effective in cases with renal involvement. Inhibition of BLyS by belimumab leads to autoregulated B-cell apoptosis and a reduction in antibodies to double-stranded forms in serum. Patients treated with belimumab in addition to standard therapy were shown to have a reduction in symptoms, a diminished frequency of disease flares, and fewer adverse events related to SLE [77]. Anifrolumab is a monoclonal antibody (IgG1 κ) targeted against the IFN-1 receptor, approved for the treatment of moderate to severe SLE without lupus nephritis. The activity of type 1 interferons, such as IFN-α, IFN-β, and IFN-κ, is involved in the pathogenesis of SLE. These type 1 interferons are inhibited by anifrolumab [78]. Rituximab has also been successfully used for SLE in patients who fail to respond to other treatments or have severe refractory SLE [79,80]. Other monoclonal antibodies show promise for future use in SLE treatment. Current clinical trials are exploring the use of itolizumab, a monoclonal antibody against CD6, and ustekinumab, a monoclonal antibody against IL-12 and IL-23, for SLE treatment [81,82]. 

Psoriatic arthritis is a chronic inflammatory arthritis present in patients with psoriasis. It is characterized by peripheral poly- or oligo-arthritis and periarticular disease such as enthesitis, or inflammation of ligament, tendon, and joint capsule attachment sites to the bone. Key cytokines involved in pathogenesis include IL-23, IL-17, and TNF-α. These cytokines ultimately lead to inflammation, bone erosion, and osteoblast production [83]. Treatment of psoriasis is dependent on the presence of skin disease as well as the presence and severity of joint involvement. Methotrexate is often used for patients with psoriasis and psoriatic arthritis, but it does not prevent long-term damage to the joints [84]. The TNF-α inhibitors infliximab and adalimumab are also approved for the treatment of severe psoriatic arthritis and are the preferred treatment over IL-17 and IL-23 inhibitors [83]. IL-17 inhibitors can be used for patients with mild-to-severe psoriatic arthritis. Secukinumab and ixekizumab are both monoclonal antibodies against IL-17 approved for the treatment of psoriatic arthritis. However, patients with inadequate responses to secukinumab have been shown to respond well when switched to ixekizumab, with a reduction in disease symptoms [85,86]. The IL-17 and IL-23 inhibitor, ustekinumab, is also used in the treatment of psoriatic arthritis and has been shown to have a similar safety profile when compared to other monoclonal antibodies, such as secukinumab, with fewer severe adverse events [87]. 

### 2.3. Antibodies Specific to Cancer 

As the challenge of finding effective small-molecule treatments for many cancers persists, monoclonal antibodies have broken onto the scene to revolutionize cancer treatment [88]. With the discovery of new molecular targets in cancer pathophysiology comes the development of new monoclonal antibody drugs. One crucial aspect of cancer growth is the tumor microenvironment [89], which entails tumor cells as well as immune cells, vasculature, and extracellular matrix. These components offer potential targets for monoclonal antibodies for treatment, especially if the tumor cells themselves are difficult to target in cases like metastasis [90]. However, other than immune checkpoints as targets [91], many clinical trials have failed to show promising efficacy [92]. 

In some cancers, like metastatic melanoma, aberrant immune cell proliferation and signaling by cancer cells cause immunosuppression, worsening the body’s ability to fight cancer. For example, melanoma cells can express PD-L1, a ligand that neutralizes immune cells by binding to their PD-1 receptor [93]. Nivolumab is a monoclonal antibody that targets PD-1, preventing its binding to the ligand, rendering the cancer’s expression of PD-L1 ineffective, and resulting in a continued immune response to the tumor [90]. Nivolumab showed increased efficacy and positive long-term outcomes when administered with other monoclonal antibodies to treat advanced melanoma, including ipilimumab and relatlimab. Ipilimumab binds the CTLA-4 receptor on the surface of T-cells, preventing immunosuppression by tumor cells and allowing the immune system to appropriately hoist an anti-tumor response [90]. Relatlimab blocks the lymphocyte-activation gene 3 (LAG-3) to also prevent immune suppression and allow for an anti-tumor response [94]. When combined with nivolumab, the length of progression-free survival significantly increased, though there was also an increased rate of treatment-related adverse effects [94]. 

Using monoclonal antibodies in techniques such as antibody-drug conjugates (ADCs) [95] and bispecific antibodies (BsAbs) [96] has become a promising avenue for cancer therapy. One widely studied ADC treatment for invasive breast cancer is trastuzumab-emtansine, a monoclonal antibody targeting the aberrant HER2 receptor conjugated to a cytotoxin that inhibits microtubules [97]. According to phase III randomized trials, trastuzumab conjugated to deruxtecan, another cytotoxic microtubule-stabilizing drug, has demonstrated a remarkable decrease in disease progression and risk of death when compared to trastuzumab-emtansine [98]. Another ADC treatment called enfortumab vedotin entails a monoclonal antibody that binds to the nectin-4 protein on bladder cancer cells and then releases conjugated monomethylauristatin E (MMAE), a microtubule disrupting agent that kills the cancer cell [99]. 

Another successful ADC is brentuximab vedotin, a CD30-specific monoclonal antibody [100] used to treat T-cell lymphoma. The addition of brentuximab vedotin to a regimen of doxorubicin, vinblastine, and dacarbazine was successful in increasing progression-free survival in patients with stage III–IV Hodgkin lymphoma in a clinical trial [101], but another clinical trial showed that a monotherapy of the monoclonal antibody pembrolizumab, a PD-1 inhibitor classically used to treat melanoma, was more effective at improving progression-free survival compared to brentuximab vedotin alone [102]. These conflicting studies show that, though there is evidence to support the inclusion of ADCs in cancer treatment, whether they are overall more efficacious than potent monoclonal antibody monotherapy requires further investigation through clinical trials. Further successes in ADCs have been limited [95], but insights show that increased levels of cytotoxic drug conjugation and increased linker stability between drug and antibody may be next for the development of successful ADCs. 

There is also research to support ADCs as effective against tumor heterogeneity [103], which has persisted as a common obstacle to cancer treatment. Tumor heterogeneity makes antibody treatment difficult, particularly for ADEPT and ADC, due to the irregular vasculature of the tumors. This feature of solid tumors causes a reduction in the efficacy of antibody therapies [104]. 

### 2.4. Antibodies for Pulmonary Diseases

There are more than 120 antibodies targeted at respiratory diseases, mainly asthma, lung cancer, and respiratory infections. While some have shown potential (like immune checkpoint inhibitors and IL-5 pathway inhibitors) and offered hope for treating respiratory diseases, others have failed clinical trials [105]. Moreover, diseases like COPD, asthma, lung cancer, and IPF are complex due to the involvement of different signaling pathways and multiple biomarkers for targeting, with no single particularly dominant cytokine or chemokine target. For this reason, a large proportion of patients with respiratory illnesses are administered one or more antibodies. It is also important to define the optimal antibody combination, route of administration, treatment duration, and biomarkers to improve efficacy in patients [105]. 

Here are several examples of these antibodies. Omalizumab against the heavy-chain constant (Cε3) domain of IgE was the first biological therapy for asthma [106]. It is also indicated in moderate-to-severe allergic asthma [105]. Ligelizumab, also targeted at the constant domain of IgE, displayed greater efficacy than omalizumab for mild asthma. Quilizumab against the M1 prime segment of IgE reduced acute asthma exacerbations. Mepolizumab and reslizumab against IL-5 were used for eosinophilic asthma and are under phase II evaluation for chronic rhinosinusitis. Its trials are also ongoing for COPD [105]. Benralizumab against IL-5Rα is indicated in severe eosinophilic asthma [107]. It has phase III trials ongoing for COPD and phase II trials ongoing for chronic rhinosinusitis [105,108]. Dupilumab against IL-4Rα is for asthma treatment and should block both IL-3 and IL-4 for therapeutic effect. It is in phase II trials for COPD [107,108]. Tezepelumab, which targetsthymic stromal lymphoproteins, is being tested and is in phase II trials for COPD [108]. Brodalumab, secukinumab, and CJM112, targeted against IL-17, were initially tested for asthma but did not show effective outcomes. They could be used to treat COPD, but this has not yet been tested. Also, no reduction in sputum neutrophil count was observed [105,108,109]. 

Respiratory syncytial virus (RSV) is another significant pulmonary pathology that can be treated by antibody therapy. RSV’s seropositivity is 100% by the time infants are 2 years old, and half of infants are admitted to having long-term sequalae. Only palivizumab, approved and marketed for RSV, has shown a reduction in hospitalizations of 55%. Apart from palivizumab, which is an antibody with a standard half-life, motavizumab, MPE8, and TLR3D3 are in the preclinical phase. MAbs with extended half-lives such as Motavizumab-YTE, suptavumab, nirsevimab, and MK-1654, are under phase trials. For some of them, the trials have been interrupted [110]. Bevacizumab and ramucirumab against VEGF and VEGF-R2 are used for non-small-cell lung cancer (NSCLC) and show benefits when used in combination with chemotherapy. Necitumumab against EGFR showed benefit as first-line therapy for advanced squamous-cell NSCLC when combined with gemcitabin and cisplatin. However, cetiximab targeted at EFGR showed conflicting results with combined therapy. Tanstmumab (Her2/Eeb2) produced a tumor response in patients with NSCLC. Seribantumab (Her3 signaling) is under testing following a positive phase III trial, but the phase III trial for patritumab (Her3) was terminated due to a lack of efficacy. Nivolumab (PD1), pembrolizumab (PD1), and atezolizumab (PDL-1) have shown survival benefits in NSCLC when combined with first-line chemotherapy. Atezolizumab is also indicated for reducing lung toxicity due to its targeting of only PDL-1. Other antibodies that have shown promise in phase trials for NSCLC include racotumomab, L19-IL-12, MABp1, and sacituzumab [105]. Panobacumab, MEDI3902, and aerucin are antibodies against *Psuedomonas aeruginosa* and inhibit biofilm formation [105,111]. MEDI3902 received a fast-track designation from the FDA in 2014 [112].

Obiltoxaximab and raxibacumab are antibodies against *Bacillus anthracis* produced after the US terrorist attacks using anthrax [105]. Lebrikizumab and tralokinumab are against IL-13 and are under phase trials for COPD and asthma exacerbations. The former is also being tested for the treatment of idiopathic pulmonary fibrosis (IPF) [105].

According to the NIH guidelines, antivirals (paxlovid and remdesevir) are the preferred treatment for COVID-19 [113]. Bebtelovimab, against spike protein, was administered to patients aged 17+ with mild to moderate COVID-19 that had the potential to progress. It is now not recommended by the FDA due to omicron resistance [114]. Tixagevimab + cilgavimab (Evusheld) against epitopes of spike protein are recommended as repeat doses every 6 months for the prevention of SARS-CoV-2 infection [115]. Balmlanivimab + etesivimab, casirvimab + imdevimab, and sotrovimab usage have been paused due to omicron variant resistance, according to NIH guidelines. One can receive real-time information about COVID-19 therapeutics by visiting the COVID network data tracker by the CDC and IDSA (https://covid.cdc.gov/covid-data-tracker/#datatracker-home, accessed on 30 June 2023). 

### 2.5. Antibodies for Cardiovascular Diseases 

Cardiovascular disease (CVD) is the leading cause of death globally, often accompanied by atherosclerosis. Atherosclerotic plaque erosion/rupture is associated with arterial occlusion (strokes/MI). The risk factors for CVD are high blood pressure, high blood cholesterol (plasma LDL level), inflammation, and diabetes. Statins are the primary treatment for high blood cholesterol but are not effective in statin-resistant patients and have limited efficacy against homozygous familial hypercholesterolemia (HoFH) [116] and statin intolerance, displaying adverse effects like myalgia and rhabdomyolysis (rare but severe) [117]. An alternative is apheresis, but this is difficult and time-consuming for patients.

For primary hyperlipidemia due to heterologous familial hypercholesterolemia (HeFH) and HoFH, statins and PCSK9 inhibitors (that function by increasing the number of LDL receptors) are less effective in treatment due to the absence of LDL receptor expression. Lomitapide and mipomersen work independently of LDL receptors but are limited by adverse effects (such as elevated ALT and hepatic steatosis). Therefore, Ab therapy can be a possible substitute for statins/apheresis [116]. 

Even if hyperlipidemia is treated, it is important to address vascular inflammation and hypertension to prevent the risk of CV events, especially if inflammation is already present [118]. A potential target for this could be ApoB-100, a protein constituent of low-density lipoprotein (LDL) and a primary driver behind plaque formation. However, no therapeutic antibodies are currently available for this. Alirocumab, an antibody against PCSK9, a serine protease that binds to LDL receptors and promotes their degradation, significantly reduced plasma LDL levels and did not affect very low-density lipoprotein (VLDL) or triglyceride (TG) levels. It is used in the treatment of atherosclerotic cardiovascular disease resistant to diet changes and statin therapy, as well as for the treatment of heterozygous familial hypercholesterolemia [119]. As of April 2021, the FDA has expanded its use to also include the treatment of homozygous familial hypercholesterolemia [120]. It has shown a reduction in CV events [121,122,123] and regression of coronary plaques, particularly when used alongside statins [124]. Evolocumab (also against PCSK9) and alirocumab share a similar safety profile, with no significant difference in efficacy. They have also shown enhanced effects in patients undergoing statin therapy. In phase III trials of bococizumab, also an antibody against PCSK9 and used for the treatment of hyperlipidemia, high levels of anti-bococizumab antibodies developed in patients, reducing overall drug efficacy. Also, an inconsistent reduction in LDL cholesterol was seen, leading to its discontinuation for further development. Evinacumab, an anti-ANGPTL3 (inhibitor of lipoprotein lipase and endothelial lipase) antibody, is involved in the degradation of circulating triglycerides and other lipids. It was developed for the treatment of HoFH. PCSK9 inhibitors and statins were less effective due to the absence of LDL receptors in patients. Evinacumab treated HoFH patients with greater success due to a mechanism of action independent of LDL receptors. Trials demonstrated a significant decrease in LDL cholesterol levels in HoFH patients, but this lacks long-term data. Canakinumab is an antibody that binds to IL-1β, helping reduce inflammation. Models suggest that reducing vascular inflammation (even without addressing cholesterol levels) should decrease the risk of CVD [118,125,126]. Canakinumab is approved for the treatment of several auto-inflammatory diseases: CAPS, TRAPS, HIDS/MKD, FMF, Still’s Disease, and SJIA. The canakinumab anti-inflammatory thrombosis outcome study (CANTOS) trial to determine efficacy against CVD showed that canakinumab was associated with a decrease in CV events but limited by its high cost and risk of fatal sepsis and therefore not approved for CVD treatment. However, it still demonstrated that inflammation—not just hyperlipidemia—was a key component of CVD [125,126]. The CANTOS trial primarily involved patients with elevated inflammatory markers, which could have affected the data. Furthermore, long-term treatment with canakinumab showed a significant reduction in incidental anemia in patients without baseline anemia. Significant increases in mean hemoglobin levels were also seen in patients with anemia [127]. IL-6 induces hepcidin production, thus demonstrating the downstream effect of IL-1 inhibition on anemia [126]. It is associated with an increased risk of infection, mild thrombocytopenia, and mild neutropenia [125,126,127]. Ziltivekimab targets IL-6 and reduces inflammation. Early trials demonstrate a reduction in inflammation without affecting the total/HDL cholesterol ratio (a common adverse effect of IL-6 inhibitors) [126]. Further trials are needed to determine the effects on CV event risk. The roles of IL-6 in angiogenesis entail the induction of VEGF, autoimmunity, and cancer [128]. Also, IL-6 induces hepcidin production, which has a role in iron homeostasis and anemia and is a factor in heart failure. Tocilizumab, an IL-6R-specific antibody that was used as a treatment for rheumatoid arthritis, is not a therapy for CVD. It is contradicted in patients with CVD risk due to a significant increase in lipid levels [129,130]. It shows interaction with CYP450, associated with atorvastatin metabolism. It is associated with pro-atherogenic increases in blood cholesterol and decreased LDL receptor expression. It has a relatively low increase in CV events compared to other bDMARDs, indicating pro-atherogenic effects possibly balanced by anti-inflammatory inhibition of IL-6 [129]. It did not show any significant treatment effect on pulmonary arterial hypertension (PAH), as no changes in vascular resistance or associated inflammatory markers were seen with the treatment response [131]. 

### 2.6. Antibodies for Renal Diseases

Rituximab, a chimeric antibody specific for the CD20 antigen on B-cells and initially approved by the FDA for lymphoma, is effective in the treatment of membranous glomerulonephritis, steroid-resistant nephrotic syndromes, and membranoproliferative glomerulonephritis (MPGN) [132]. Clinical studies on rituximab support it as an effective steroid-sparing agent in steroid-dependent idiopathic nephrotic syndrome [133]. In April 2011, it was approved for the treatment of anti-neutrophil cytoplasmic antibody (ANCA)-associated vasculitis (including granulomatosis with polyangiitis (Wegener’s granulomatosis) and microscopic polyangiitis) [134]. Additionally, it is used in the treatment of membranous nephropathy and lupus nephritis [134]. Minimal change disease (MCD) or focal segmental glomerulosclerosis (FSGS) are two major causes of nephrotic syndrome and are treated with steroids, antiproteinuric drugs, and immunosuppressants, as suggested by the KDIGO (Kidney Disease: Improving Global Outcomes) guidelines. The monoclonal antibodies shown to have a positive result in reducing proteinuria in small cohorts are rituximab, ofatumumab, adalimumab, fresolimumab, and bleselumab. Out of these, rituximab’s effects were established by larger trials, while other antibodies like ofatumumab, fresolimumab, and adalimubab showed conflicting results in novel therapies for resistant FSGS clinical trials (FONT) and FONT II (phase II) trials. The effectiveness of bleselumab on post-transplant FSGS is currently under investigation [135]. Rituximab is also instrumental in reducing the auto-antibody formation in ANCA-associated vasculitis, lupus nephritis, and mixed cryoglobulinemia. As mentioned earlier, it has been shown to safely decrease proteinuria in patients with nephrotic syndrome secondary to membranous nephropathy, MGS, and FSGS. Eculizumab, an anti-C5 humanized monoclonal antibody, is also documented to be efficient in treating C3 nephropathy, atypical hemolytic uremic syndrome, and membranoproliferative glomerulonephritis [136]. Other antibodies, such as adalimumab, daclizumab, fresolimumab, belimumab, and tocilizumab, some of which are already approved for different medical applications, are under premarketing investigation [132]. Apart from their positive effects, some antibodies also cause renal toxicities. Examples include hypertension and proteinuria caused by bevacizumab, an anti-VEGF antibody, along with other anti-VEGF therapeutics like aflibercept (VEGF trap) and anti-VEGF receptor (VEGFR) tyrosine kinase inhibitors (TKIs) [137]. Cetuximab and panitumumab, monoclonal antibodies against the HER-family of receptors, caused electrolyte imbalances, including hypomagnesemia and hypokalemia, due to the direct nephrotoxic effect of the drug on renal tubules. Cetuximab displayed potential for renal tubular acidosis. It was also mentioned that rituximab caused acute renal failure following the initiation of therapy due to the onset of acute tumor lysis syndrome. Therefore, it is important to discern the adverse effects of the therapeutics to ensure safe treatment strategies, especially in patients with pre-existing renal conditions [137]. 

### 2.7. Antibodies for Gastrointestinal Pathologies 

While the exact pathogenesis of inflammatory bowel disease (IBD) remains under investigation, aberrant cytokine release is known to play a significant role in the progression of both Crohn’s disease and ulcerative colitis [138,139]. In patients with IBD, elevated levels of TNFα have been associated with inflammatory tissue damage and impaired regulation of intestinal immune cells [138,140,141,142]. Two TNFα inhibitors, infliximab and adalimumab, have been approved by the FDA for the treatment of moderate-to-severe Crohn’s disease and ulcerative colitis [143]. 

Infliximab, a chimeric mouse/human antibody, binds soluble and transmembrane TNFα, inhibiting its pro-inflammatory activity and inducing lysis of TNFα-producing cells [143,144]. Infliximab has been found to induce mucosal healing and maintain remission of both Crohn’s disease and ulcerative colitis, with a promising safety profile for long-term treatment [145,146,147,148,149]. Adalimumab, a monoclonal human antibody, neutralizes the biological activity of TNFα in a similar manner and has comparable effectiveness in treating IBD [150,151,152,153,154,155,156,157]. Patients treated with infliximab or adalimumab may develop anti-drug antibodies and a resultant loss of response to mAb therapy [158,159]. Switching from one TNFα inhibitor to another was able to restore treatment response in some patients [160,161]. Combination therapy with an immunomodulator (thiopurines or methotrexate) has also been found to improve initial drug efficacy, decrease immunogenicity, and recapture treatment response [162,163,164,165,166]. Immunomodulator use remains controversial, however, due to its association with the development of severe infections and malignancies [148,167,168].

Certolizumab pegol is an anti-TNFα fragment conjugated to polyethylene glycol, humanized to reduce immunogenicity and the development of anti-drug antibodies [169,170,171,172]. This drug was FDA-approved for use in the treatment of Crohn’s disease [173]. PEGylation of certolizumab increases the elimination half-life and possibly further decreases immunogenicity [174,175,176]. Golimumab is another anti-TNFα mAb recently approved for the treatment of ulcerative colitis only [177,178]. Clinical trials and early studies have shown golimumab to be safe and have similar effectiveness to infliximab and adalimumab [179,180]. Both certolizumab pegol and golimumab appear to have lower immunogenicity and decreased anti-drug antibody levels relative to infliximab and adalimumab [181,182].

Beyond TNFα inhibitors, anti-integrin mAb therapy is another promising avenue for IBD treatment. Natalizumab binds α4 integrin, blocking α4β7-integrin/mucosal addressin cell adhesion molecule-1 (MadCAM-1) interactions and α4β1/vascular-cell adhesion molecule-1 (VCAM-1) binding [183,184]. By inhibiting these interactions, natalizumab prevents immune-cell migration to the inflamed intestinal mucosa, preventing further tissue damage. Similarly, vedolizumab limits leukocyte homing to the gut mucosa [185] by directly binding and neutralizing α4β7. Natalizumab is typically reserved as a second-line treatment following failure of TNFα inhibitor treatment due to the risk of progressive multifocal leukoencephalopathy from the reactivated John Cunningham polyoma virus [186]. While both mAbs are approved for the treatment of Crohn’s disease, vedolizumab is additionally approved for ulcerative colitis [187]. In a head-to-head trial involving patients with moderate to severe ulcerative colitis, vedolizumab was found to have higher efficacy than adalimumab [185]. Etrolizumab (anti-β7 subunit of α4β7 and αEβ7 integrins) remains under study for the treatment of IBD. While abandoned as an ulcerative colitis treatment due to mixed phase III trial results, clinical trials involving patients with Crohn’s disease demonstrate high safety and efficacy at remission maintenance [188,189]. 

With the continued success of mAb IBD treatments, therapeutic antibodies have been additionally explored for other gastrointestinal conditions. Aberrant leukocyte trafficking may be involved in the pathogenesis of IBD-associated primary sclerosing cholangitis (PSC) [190]. Anti-integrin mAbs have been considered for PSC treatment, although vedolizumab treatment failed to provide a clinical benefit to patients [190,191,192,193,194]. Lysyl oxidase like-2 (LOXL2) is known to drive fibrogenesis and is also believed to be involved in the progression of PSC and non-alcoholic steatohepatitis (NASH). Simtuzumab, an antibody directed against LOXL2, was found to be ineffective in alleviating PSC liver damage [195,196]. The monoclonal antibody CM-101 offers a promising therapy for NASH and liver fibrosis. CM-101 selectively targets CCL24, a chemokine involved in mediating inflammation and fibrotic processes, and has demonstrated a significant reduction in liver fibrosis in animal models [197]. Phase II trials are currently ongoing (NCT04595825).

Mepolizumab and reslizumab, anti-IL5 antibodies approved for the treatment of eosinophilic asthma, have been investigated for the treatment of eosinophilic esophagitis. Although IL-5 inhibition suppressed eosinophil accumulation, symptom improvement was inconsistent and not clinically significant [198]. Other pathogenic mechanisms may be involved in the progression of eosinophilic esophagitis beyond IL-5-driven eosinophilia. 

### 2.8. Antibodies for Infectious Diseases 

The COVID-19 global pandemic showed the effect of infectious disease on our increasingly connected world, reemphasizing and reprioritizing the focus of infectious diseases for pharmaceutical treatment. COVID-19 caused an estimated 921,000 deaths [199] and cost an estimated USD 163 billion [200] just in the United States. However, globally, the top three infectious diseases (HIV, tuberculosis, and malaria) have contributed to an economic burden of up to USD 7 trillion and over 140 million years of life lost [201]. In response, there has been considerable interest in developing new methods to both prevent the spread of infectious diseases and contain them after infection, especially with the use of monoclonal antibodies.

Derived from humanized IgG1, palivizumab was the first FDA-approved monoclonal antibody treatment for infectious disease—specifically for respiratory syncytial virus (RSV). Palivizumab binds and prevents key conformational changes in the viral F protein, preventing virus attachment and fusion. RSV is a common cause of lower respiratory tract infections, and severe cases require hospitalization, making RSV a significant cause of morbidity for young children [202]. Despite significant efforts [203,204], no vaccines have been made available for RSV, making palivizumab the only prophylaxis for RSV. It has also been effective at reducing hospitalization due to RSV infection [205,206]. Despite its high cost and requirement to be administered every month for five months during RSV season, it has been shown to be cost-effective in patients where treatment is indicated [207].

Another advantage of monoclonal antibody therapy development for infectious diseases is the ability to isolate human immunoglobulins from infected patients to study therapeutic effects or to directly use in treatment. Ansuvimab, an FDA-approved human IgG1 antibody to treat Ebola virus infections, was initially isolated from a survivor of a 1995 Ebola outbreak [208]. The drug binds and inhibits the Ebola virus glycoprotein and the Niemann-Pick C1 (NPC1) receptor, preventing viral membrane fusion and entry. Inmazeb, a cocktail of three fully human monoclonal antibodies obtained from VelocImmune mice, has also been approved to treat Ebola virus infections [209] and has demonstrated protection against escape mutants [210].

Thus far, only seven monoclonal antibodies have been FDA-approved for infectious diseases. In addition to the aforementioned drugs, these include raxibacumab and obiltoxaximab for the treatment of anthrax infection, bezlotoxumab for the prevention of *Clostridiodes difficile* infection recurrence, and ibalizumab for the treatment of HIV-1 infection [211]. Research into the use of monoclonal antibodies in infectious diseases is ever-growing, and future directions of development are aimed at its integration into vaccine development and the treatment of previously untreatable infections [212].

### 2.9. Antibodies for Endocrine Disorders 

Type 1 diabetes mellitus (T1DM) is an endocrine disease driven by autoimmune destruction of pancreatic β-cells [213,214,215]. The progressive loss of these insulin-producing β-cells results in a declining ability to moderate serum glucose levels. In patients with T1DM, insulin depletion and the resulting chronic elevation of blood glucose are associated with the development of atherosclerosis, neuropathy, and retinopathy [216].

Teplizumab, an anti-CD3 mAb, is currently the only disease-modifying therapy approved for T1DM [217]. By targeting CD3, Teplizumab blocks T-cell receptor interactions and inhibits T-cell activation, an important component of the autoimmune cell-mediated tissue damage in T1DM [218,219]. Anti-CD3 therapy has been associated with partial exhaustion of T-effector cells and an induction of regulatory T-cell activity [218,220,221,222,223]. Treatment has been shown to preserve β-cell function in new-onset T1DM [224,225], significantly slow the long-term progression of overt T1DM [226,227], and delay the onset of T1DM in high-risk individuals [223,228]. Similarly, another anti-CD3 mAb called otelixizumab has been found to protect β-cell function in patients with T1DM and appears safe [229,230,231].

Additional T-cell-targeted therapies include anti-thymocyte globulin (ATG), a polyclonal antibody directed against multiple T-cell antigens. High-dose (6.5 mg/kg) ATG monotherapy was ineffective in preserving β-cell function, although this result was likely due to generalized T-cell depletion without sparing regulatory T-cells [232,233]. Administration of a lower dose of ATG with granulocyte colony stimulating factor (GCSF) appears to deplete T-effector cells without significant reductions in regulatory T-cell levels [234,235]. Low-dose ATG/GCSF combined therapy slowed the decline of C-peptide in individuals with new-onset and established T1DM, indicating preserved β-cell function. However, low-dose ATG monotherapy appears to be more efficacious than low-dose ATG/GCSF, while GCSF alone is ineffective in maintaining β-cell function [236,237,238,239]. Phase II trials are currently ongoing to further evaluate low-dose ATG as a disease-modifying treatment for T1DM (NCT04291703 and NCT04509791).

Rituximab, an anti-CD20 antibody causing B-cell depletion in non-Hodgkin’s lymphoma, chronic lymphocytic leukemia, and rheumatoid arthritis, provides an additional candidate for T1DM treatment. Reflecting the secondary role of autoantibodies in the progression of T1DM, rituximab-mediated B-cell depletion was found to decrease HbA1c, reduce insulin requirements, and slow the decline of C peptide levels in patients with T1DM [240]. Although unable to maintain remission over the long term and overall less effective than teplizumab or low-dose ATG [239,241], rituximab may have greater success when used in a combined therapy. Autologously expanded regulatory T-cells and rituximab were found to have significantly greater efficacy compared to either treatment as a monotherapy.

### 2.10. Antibodies for Neurological Disorders

Antibody-based therapeutics have proven to be beneficial and are used as a standard in treating a spectrum of neurological and neuroinflammatory diseases. Multiple sclerosis (MS) is a chronic, multifactorial neurological condition that affects the central nervous system via autoimmune-mediated inflammation. Activated T-cells cause demyelination [242] of neurons, leading to clinical symptoms like fatigue, pain, depression, and anxiety. Natalizumab (see Section 2.7, Gastrointestinal) prevents the extravasation of these activated T-cells by binding to integrins, which are leukocyte adhesion molecules [243]. The inhibition of leukocyte extravasation reduces immune-mediated inflammation and slows the progression of MS [244]. This is particularly useful for the long-term management of MS [245]. Though the safety and efficacy of natalizumab have been shown, there has been significant study into one of the main adverse effects of natalizumab treatment—progressive leukoencephalopathy [246]. Current efforts are devoted to identifying, quantifying, and mitigating the risks associated with natalizumab treatment [247]. 

Other monoclonal antibodies also used to treat MS include alemtuzumab, daclizumab, rituximab, ocrelizumab, and ofatumumab [248]. Alemtuzumab binds the CD52 receptor of lymphocytes, causing lymphocyte lysis and depletion. Daclizumab binds the receptor IL-2R α to prevent IL-2 signaling-mediated inflammation. Rituximab, ocrelizumab, and ofatumumab all bind the CD20 receptor of B cells, leading to cell depletion. All these monoclonal antibodies treat relapsing-remitting multiple sclerosis (RRMS), which is characterized by intervals of symptom remission followed by intervals of severe symptom resurgence. 

A major target of monoclonal antibody drug development has been the amyloid-β peptide (Aβ). Alzheimer’s disease is characterized by Aβ aggregates and neurofibrillary tangles in the brain, accompanied by synaptic dysfunction and neurodegeneration. Several antibodies have been developed targeting both the soluble and aggregated forms of the Aβ peptide [249]. Reductions of biomarkers found in Alzheimer’s disease have been shown in clinical trials for monoclonal antibodies but have only minimally improved clinical outcomes in patients. The risk of amyloid-related imaging abnormalities with edema remains a significant risk of these drugs, especially in APOE4 carriers [250]. However, current drugs used for the treatment of Alzheimer’s, such as cholinesterase inhibitors, treat only symptoms and do not show any reduction in the clinical progression of the disease [251]. Due to the pathological complexity of Alzheimer’s disease, further studies are recommended for the use of monoclonal antibodies. 

Aducanumab is a human monoclonal antibody (IgG1) that selectively binds to and reduces the soluble and insoluble forms of Aβ found in the brain. Animal studies have shown Aducanumab to be more effective in preventing Aβ aggregation than clearing existing aggregates in the brain, possibly limiting the ability of the drug to improve rather than just slow cognitive impairment [252]. Current phase III clinical trials for Aducanumab have shown a reduction in Aβ peptides in CSF and plasma in Alzheimer’s patients, but further studies are needed to confirm a reduction in clinical decline [253,254]. Lecanemab is a humanized monoclonal antibody (IgG1) that selectively binds to soluble Aβ protofibrils. However, the risk of amyloid-β-related edema limits the strength of the dose used in patients with the ApOE4 allele. In phase II clinical trials, lecanemab was shown to remove Aβ aggregates from the brain, leading to a reduction in cognitive impairment symptoms and diminished Alzheimer’s CSF biomarkers [255]. Bapineuzumab is also a humanized monoclonal antibody (IgG1) against insoluble Aβ that has shown changes in amyloid-related imaging and concentrations of cerebrospinal fluid phospho-tau in carriers of the ApOE4 allele only. However, improvement in clinical outcomes for carriers and non-carriers has not been shown in phase III trials [256,257]. Additional monoclonal antibody candidates for the treatment of Alzheimer’s disease include ponezumab, crenezumab, and gantenerumab [255,258,259]. 

Parkinson’s disease is a neurodegenerative disorder caused by the aggregation of α-synuclein, which is a neuronal protein integral to presynaptic vesicle trafficking [260]. Prasinezumab is a monoclonal antibody targeted at aggregated α-synuclein. Recent studies have demonstrated no significant therapeutic effect of prasinezumab [261] when compared to placebo groups and, in fact, resulted in infusion site reactions. This has left Parkinson’s disease as a condition that has not yet been successfully treated by monoclonal antibody therapy. 

Although often disregarded by many as a true illness [262], chronic migraine is a neurological condition characterized by a severe headache that affects one in nine adults globally [263]. Notably, migraines have arisen as a goal for antibody therapy by targeting calcitonin gene-related peptide (CGRP). Erenumab, fremanezumab, and galcanezumab have all been approved as humanized monoclonal antibody drugs to treat migraines [262]. Though erenumab showed success in significantly reducing the frequency of migraines, its long-term safety has yet to be tested [264]. Similarly, a phase III, randomized clinical trial demonstrated that galcanezumab was significantly more effective at reducing the number of days with migraines per month while also being well tolerated [265]. Fremanezumab also showed decreased headache frequency over a 12-week trial, though patients also presented with injection-site reactions [266]. Nonetheless, fremanezumab was well tolerated and resulted in sustained migraine improvement for up to 12 months in patients, according to a randomized study [267]. An additional meta-analysis of randomized controlled trials also confirmed that fremanezumab was efficacious but was accompanied by only mild adverse effects in some patients [268]. 

### 2.11. Antibodies for Ophthalmological Disorders

Ranibizumab is a monoclonal antibody that can treat retinopathy of prematurity (ROP). Ranibizumab targets VEGF-A and is administered via injection to the vitreous humor of the eye. It has shown success at 24 weeks in infants with ROP in a randomized phase III trial. Furthermore, it does not seem to cause systemic suppression of vascular endothelial growth factor, though long-term effects on vision are yet to be studied [269]. 

Bevacizumab was proposed as another monoclonal antibody that can treat ROP, but a systematic review and meta-analysis of thirteen studies suggested that treatment can cause an increased risk of cognitive impairment and a decreased language score in infants [270]. Naxitamab is an anti-cancer drug used with GM-CSF to treat relapsed or refractory neuroblastoma. Notably, the FDA approved a combined naxitamab-GM-CSF (granulocyte-macrophage colony-stimulating factor) therapy to treat refractory neuroblastoma in pediatric patients about the age of one and in adult patients [271]. 

### 2.12. Antibodies for Musculoskeletal Disorders

Musculoskeletal diseases also have great success with monoclonal antibody therapy, largely due to a better understanding of the molecules and pathways involved in musculoskeletal metabolism and regulation. RANK-L is a membrane-bound ligand present on osteoblasts, and it is important for bone turnover. Denosumab is a human IgG2 that binds and inhibits RANK-L, preventing its interaction with the RANK receptor. This inhibition blocks the maturation and function of osteoclasts, resulting in reduced bone resorption [272], making denosumab a suitable treatment for osteoporosis, especially in postmenopausal women at increased risk of bone fractures. 

Romosozumab is a humanized IgG2 antibody that binds sclerostin, another key molecule in the bone remodeling pathway expressed by osteocytes. Inhibition of sclerostin by romosozumab inhibits bone resorption and promotes bone formation, allowing romosozumab to also be used as a treatment for osteoporosis in postmenopausal women [273]. According to a systematic review and meta-analysis of randomized controlled trials, romosozumab resulted in a statistically significant reduction in the incidence of vertebral and non-vertebral fractures, as well as a statistically significant increase in bone mineral density of the lumbar spine, hip, and femur [274]. A study comparing denosumab with the newer romosozumab showed that the romosozumab group experienced a significant increase in mean bone mineral density over 12 months when compared with the denosumab group [275]. These differences were present not only in bone mineral density at the lumbar spine but also for the total hip and femoral neck, suggesting that romosozumab may have a higher potential to improve bone mineral density in women with postmenopausal osteoporosis. Burosumab is a human IgG1 that targets fibroblast growth factor 23 to treat X-linked hypophosphatemia in children [276] and adults [277]. A placebo-controlled phase III study demonstrated burosumab’s efficacy in improving stiffness, pain, and fatigue [278]. 

Though an autoimmune condition [279], myasthenia gravis is a disease presenting with muscle weakness due to aberrant signaling at the neuromuscular junction. In evaluating available treatments, a network meta-analysis showed that eculizumab, a humanized IgG2/4 that binds C5 of the complement cascade, was the most effective and well-tolerated therapy to treat refractory myasthenia gravis when compared to tacrolimus, cyclosporine A, and mycophenolate mofetil. 

All the sections listed in Section 2 are summarized in Table 1, which provides an organized overview of antibodies implicated in treating various diseases in different branches of medicine [47].

## 3. Critical Analysis and Adverse Effects

Although mAbs have been implicated in the treatment of various diseases, their use has been associated with immune-mediated adverse reactions, despite continuous efforts to humanize the antibodies. Apart from immune reactions, there have been incidences of an increase in infections and a potential risk for malignancies and secondary autoimmunity. Some of the adverse effects can be predicted by the target specificity and mechanism of action; however, some remain unpredictable (e.g., hepatotoxicity associated with natalizumab) [415]. The immune-related manifestations of mAb treatment include infusion-related reactions (causing a range of symptoms from rash to generalized edema and cardiac arrest), anaphylactic reactions, and complement activation-related pseudo-allergy (CARPA), as was seen in rituximab and infliximab [416,417].

Mabs have also been seen to activate T-cells, causing cytokine release syndrome (CRS). Severe, life-threatening CRS was observed while treating hematological malignancies with rituximab and alemtuzumab [418]. Therefore, prophylactic protocols that include corticosteroids to prevent CRS are used in the cases of rituximab, ocrelizumab, and alemtuzumab. Mabs are occasionally identified as allogenic, resulting in an immunogenic response that tends to neutralize and rapidly eliminate them from the body via the development of anti-drug antibodies (ADA). This leads to allergic reactions, reduced efficacy, and an increased cost of treatment. Chimeric mAbs like infliximab and adalimumab are shown to be more immunogenic, leading to the formation of ADAs, than human mAbs. Examples include natalizumab, an anti-CD49d antibody that resulted in ADAs in 9% of multiple sclerosis (MS) patients, with 6% of patients developing permanent ADAs, as indicated by the high ADA titers [419]. This can be compared to results in the case of alemtuzumab (for B-cell lymphocytic leukemia), with which 29% of patients in CARE-MS I/II showed development of ADAs in serum after one year without loss of efficacy, and erenumab (human anti-CGRP receptor antibody), with which 2–8% of patients developed ADAs without any reduction in efficacy or increased incidence of adverse events [420,421,422].

Mabs that affect immune function by reducing cell populations (e.g., rituximab and ocrelizumab) or blocking cell migration through the endothelium (e.g., natalizumab) have shown associations with opportunistic infections. Treatment with natalizumab was associated with cryptococcal meningitis and activation of latent tuberculosis (TB) [423]. Alemtuzumab has also shown reactivation of latent TB. The development of progressive multifocal leukoencephalopathy (PML) was reported with natalizumab, rituximab, and ocrelizumab. Alemtuzumab and ocrelizumab were also linked to a significant increase in the overall risk of infection [415].

There is also a likelihood of developing malignancy due to the immunosuppressive effects of antibodies. In the phase III trial of progressive MS, 11 cases of malignancy were reported for ocrelizumab, four of which were breast adenocarcinomas. Therefore, women on ocrelizumab are advised to undergo standard breast cancer screenings, according to the European Medicines Agency’s public assessment report on Ocrevus (brand name for ocrelizumab) [424]. However, rituximab, on the other hand, did not show an increase in the incidence of cancer. The potential risk of immunosuppression and immunocompromising effects of mAbs creates a need for further evaluation. Nonetheless, the overall risk-benefit balance of approved mAbs is not significantly impacted.

Mabs specific for immunologic epitopes were seen to be associated with various autoimmune diseases. In 2018, daclizumab was withdrawn due to the development of secondary autoimmune conditions against the central nervous system, liver, and skin [425]. Alemtuzumab was also associated with the development of secondary autoimmunity, with up to 29% of patients developing thyroiditis, followed by idiopathic thrombopenic purpura (ITP) and Goodpasture syndrome. Other autoimmune disorders related to alemtuzumab use are diabetes mellitus type I, autoimmune hemolytic anemia, Still’s disease, myositis, vitiligo, and alopecia areata universalis [415].

The adverse events explain the failure of the treatment of diseases with antibodies. Since adverse reactions are associated with treatment administration and their evolution after treatment discontinuation, safety can only be guaranteed by the development of clinical programs and post-marketing pharmacovigilance monitoring [415]. 

## 4. Future Directions of Antibody Therapeutics

Technological advancements in drug development and research opened the doors for numerous strides in antibody design. Several methods to develop antibodies have emerged, including antibody conjugates (e.g., ADC, radioimmunoconjugate, ADEPT, etc.), bispecific or multispecific antibodies, and antibody fragments (scFvs, Fab, VHH, etc.). Antibody engineering strategies can be employed to confer improved affinity for targets and develop multi-targeted antibodies that can simultaneously treat multiple diseases. Even the adjustment of antibody variable domains can be conducted to target molecules characterized by genetic or genomic differences. Bispecific/multispecific antibodies, ADCs, and CAR-T are all emerging as potential antibody therapeutics. Promising approaches include engineering antibodies with one tumor-cell-specific antigen binding domain and one immune-cell-specific antigen binding domain to ideally stimulate the immune response to cancer cells [426].

To make therapeutic antibodies more effective, miniaturization and multi-functionalization are two important antibody development strategies [427]. While the engineered fragmented antibodies offer advantages such as better tissue penetration and enhanced serum clearance that make them suitable candidates for localized targeting and diagnostic applications, multi-functionalization (tagging with a drug/payload) helps with improved biodistribution profiles. Making the antibodies multispecific helps improve the effector function, as it assists with T-cell co-stimulation and recruitment of adaptive and innate immune cells, immune checkpoint blockade, or multiple antigen targeting [427]. 

Developing personalized antibody-based vaccines can be another avenue to explore in the future. The resources offered by modern advances like deep sequencing, single-cell RNA sequencing, second-generation sequencing, and spatial omics, along with integrated bioinformatics, provide us with the ability to have a comprehensive understanding of diseased cells. This provides an opportunity to venture into and develop novel antibody-based therapeutics for intracellular targeting and precision medicine [427].

The future of antibody therapeutics should also entail the optimization of antibodies to target a wide range of diseases, from cancer to infectious diseases. Though several antibody therapies have been developed for such conditions, an enduring difficulty in immunoglobulin therapy is the delivery of antibodies across biological barriers [428]. Though there is increased literature supporting the development of nanobodies, or smaller antibodies that can better permeate biological barriers, there is still potential for improved delivery of full-size antibodies via bio-conjugation to other molecules or liposomes as a delivery method [429,430,431]. The necessity to administer antibodies intravenously, intramuscularly, and subcutaneously has also evolved, as even intranasal methods are being researched as non-invasive means of antibody drug delivery [432]. Such improvements in delivery can increase the ability of antibody drugs to treat conditions affecting the brain and lungs.

Newer applications of machine learning and artificial intelligence (AI) are improving and increasing the efficiency of antibody therapy discoveries through the identification of common motifs and epitopes. Computational analysis of antibody chemical structures or physical properties can be accomplished in a way that is more efficient than time-intensive methods like X-ray crystallography [433]. Structural prediction of antibody elements like complementarity-determining regions has improved, but de novo design is a persisting challenge [433]. 

Recent studies have shown that data-trained machine learning tools can be utilized alongside antibody repertoire libraries like the observed antibody space (OAS) to efficiently design new antibodies. Since humanization of murine antibodies remains the predominant method of monoclonal antibody drug synthesis [434], these tools generate and evaluate the humanness of antibodies to reduce adverse immunogenicity reactions while also providing necessary diversity to sequences. Hu-mAb is one such novel computational tool that suggests mutations to reduce immunogenicity, and it has shown that it can produce results similar to those of experimentally deduced mutations yet far more efficiently [435]. AntiBERTa is another such tool that has experimentally outperformed manual and other AI methods of antibody design [436]. More recently, BioPhi is another such computational tool that has novel methods for both humanization (named Sapiens) and evaluation of humanness (named OASis) [434]. Another study used one such design model and further applied an artificial intelligence-based model to generate binders and characterize them by surface plasmon resonance, elucidating three binders with higher binding affinity than the known therapeutic antibody trastuzumab [437]. According to Li et al. (2023) [438], the combination of several different tools like RaptorX, DeepH3, DeepAb, DeepSCAb, ABlooper, Rosetta, and AbAdapt enabled teams to optimize monoclonal antibody design on several levels: prediction of whole antibody variable regions, prediction of antibody rotamer geometry, prediction of rigid docking between antibody and antigen, and even de novo prediction of CDR-H3 loop sequences and structures, which addresses a challenge of AI-powered antibody design mentioned by Kim et al. (2023) [433]. A study published in June 2023 [438] emphasized the combination of tools like Prihoda’s BioPhi with tools like solPredict in order to optimize for antibody tendency to aggregate, solubility, and pharmacokinetics. Such methods are instrumental in the future of high-throughput monoclonal antibody drug production [434,435,437,439]. Furthermore, Hie and coworkers demonstrated the potential of language-model-guided affinity maturation that enhanced the binding affinities of four clinically relevant antibodies and unmatured antibodies by several folds, along with thermostability and viral neutralization activity against Ebola and SARS-CoV-2 pseudo-viruses [440].

A particular focus of AI-supported antibody engineering is the challenge of reducing the immunogenicity of therapeutics, especially in the field of cancer immunotherapy [438]. The AI-powered model used in this study was able to identify and analyze peptide sequences optimized not only for peptide-MHC binding but also for reduced immunogenicity, decreasing the likelihood of ADA formation. Additionally, the study’s analysis of immunotherapeutic responses notably entailed the detection of tumor-specific biomarkers to determine the clinical appropriateness of cancer immunotherapy. This offers a great insight into the potential of AI to transform oncological treatment. Though there is still progress to be made in terms of the volume of shared data that can improve AI and ML models, the integration of AI in a clinical capacity is inevitable for physicians seeking monoclonal antibody therapies for cancer [438].

## 5. Conclusions

Therapeutic antibodies have emerged as a hot spot in the area of drug development for the treatment of autoimmune diseases, malignant tumors, infectious diseases, and neurological disorders. The rapid expansion of the antibody industry in different therapeutic areas is indicative of its virtually limitless potential. Not only are they beneficial to patients, but they also generate significant revenue for companies invested in antibody therapeutic research and development. However, it is also important to acquire an understanding of their indications, potential side effects, and risk profiles to minimize adverse reactions. Given the myriad of projects completed in the field, future approaches should aim to design novel molecules based on the unmet needs of patients. This can be conducted by assessing clinical needs, investing more in drug discovery and disease mechanism research for continued development, and improving in the field of therapeutic antibody development. With the plethora of resources offered by technological advancements and the application of AI in biomedicine, future antibody therapeutics hold tremendous potential to become a mainstay in the space of personalized medicine.

## Figures and Tables

**Table 1:** System-wise list of therapeutic antibodies specific to different targets implicated in different branches of medicine (adapted from the antibody society website) [47]. Asterisk (*) indicates country-specific approval, hash sign (#) indicates that they are either withdrawn or marketing has been discontinued for the first approved indication. NA indicates that they are either not approved or are in review in the EU or not approved/information on review status is not available in the US.

Therapeutic Area	INN	Brand Name	Target	Format	Use	Ref	US Approval Year	EU Approval Year
Hematologic	Concizumab	Alhemo™	Tissue factor pathway inhibitor	Humanized IgG4	Hemophilia	[280]	Review	Review
Hematologic	Sutimlimab	Enjaymo	C1s	Humanized IgG4	Cold agglutinin disease	[281]	2022	2022
Hematologic	Emicizumab	Hemlibra	Factor IXa, X	Humanized IgG4, bispecific	Hemophilia A	[58]	2017	2018
Hematologic	Crizanlizumab	Adakveo	P-selectin	Humanized IgG2	Sickle cell disease	[64]	2019	2020
Hematologic	Caplacizumab	Cablivi	von Willebrand factor	Humanized Nanobody	Acquired thrombotic thrombo- cytopenic purpura	[282]	2019	2018
Hematologic	Ravulizumab	Ultomiris	C5	Humanized IgG2/4	Paroxysmal nocturnal hemoglobinuria	[283]	2018	2019
Hematologic	Daratumumab	Darzalex	CD38	Human IgG1	Multiple myeloma	[284]	2015	2016
Hematologic	Elotuzumab	Empliciti	SLAMF7	Humanized IgG1	Multiple myeloma	[285]	2015	2016
Hematologic	Idarucizumab	Praxbind	Dabigatran	Humanized Fab	Reversal of dabigatran-induced anticoagulation	[286]	2015	2015
Hematologic	Eculizumab	Soliris	C5	Humanized IgG2/4	Paroxysmal nocturnal hemoglobinuria	[287]	2007	2007
Hematologic	Rituximab	MabThera, Rituxan	CD20	Chimeric IgG1	Non-Hodgkin lymphoma	[288]	1997	1998
Hematologic	Elranatamab	(Pending)	BCMA, CD3	Humanized IgG2	Multiple myeloma	[289]	Review	Review
Hematologic	Talquetamab	(Pending)	G protein-coupled receptor 5D, CD3	Humanized IgG4 bispecific	Multiple myeloma	[290]	Review	Review
Hematologic	Epcoritamab	EPKINLY™	CD20, CD3	Bispecific humanized IgG1	Diffuse large B-cell lymphoma	[291]	2023	Review
Hematologic	Glofitamab	(Pending)	CD20, CD3e	Bispecific 2 + 1 IgG1 CrossMab	Diffuse large B-cell lymphoma	[292]	2023	Pos Opinion
Hematologic	Alemtuzumab	MabCampath, Campath-1H; Lemtrada	CD52	Humanized IgG1	Multiple sclerosis	[293]	2014	2013
Hematologic	Ublituximab	BRIUMVI	CD20	Chimeric IgG1	Multiple sclerosis	[294]	2022	2023
Hematologic	Teclistamab	TECVAYLI	BCMA, CD3	Humanized bispecific IgG4	Multiple myeloma	[295]	2022	2022
Hematologic	Mosunetuzumab	Lunsumio	CD20, CD3	Humanized bispecific IgG1	Follicular lymphoma	[296]	2022	2022
Hematologic	Mogamulizumab	Poteligeo	CCR4	Humanized IgG1	Cutaneous T-cell lymphoma	[297]	2018	2018
Hematologic	Ocrelizumab	OCREVUS	CD20	Humanized IgG1	Multiple sclerosis	[298]	2017	2018
Hematologic	Ofatumumab	Arzerra	CD20	Human IgG1	Chronic lymphocytic leukemia	[299]	2009	2010#
Hematologic	Loncastuximab tesirine	Zynlonta	CD19	Humanized IgG1 ADC	Diffuse large B-cell lymphoma	[300]	2021	2022
Hematologic	Belantamab mafodotin	BLENREP	BCMA	Humanized IgG1 ADC	Multiple myeloma	[301]	2020	2020
Hematologic	Tafasitamab	Monjuvi, Minjuvi	CD19	Humanized IgG1	Diffuse large B-cell lymphoma	[302]	2020	2021
Hematologic	Isatuximab	Sarclisa	CD38	Chimeric IgG1	Multiple myeloma	[303]	2020	2020
Hematologic	Polatuzumab vedotin	Polivy	CD79b	Humanized IgG1 ADC	Diffuse large B-cell lymphoma	[304]	2019	2020
Hematologic	Moxetumomab pasudotox	Lumoxiti	CD22	Murine IgG1 dsFv immunotoxin	Hairy cell leukemia	[305]	2018	2021
Hematologic	Inotuzumab ozogamicin	BESPONSA	CD22	Humanized IgG4, ADC	Hematological malignancy	[306]	2017	2017
Hematologic	Blinatumomab	Blincyto	CD19, CD3	Murine bispecific tandem scFv	Acute lymphoblastic leukemia	[307]	2014	2015
Hematologic	Obinutuzumab	Gazyva	CD20	Humanized IgG1; Glycoengineered	Chronic lymphocytic leukemia	[308]	2013	2014
Hematologic	Brentuximab vedotin	Adcetris	CD30	Chimeric IgG1, ADC	Hodgkin lymphoma, systemic anaplastic large-cell lymphoma	[309]	2011	2012
Hematologic	Tositumomab-I131	Bexxar	CD20	Murine IgG2a	Non-Hodgkin lymphoma	[310]	2003 #	NA
Hematologic	Ibritumomab tiuxetan	Zevalin	CD20	Murine IgG1	Non-Hodgkin lymphoma	[311]	2002	2004
Hematologic	Gemtuzumab ozogamicin	Mylotarg	CD33	Humanized IgG4, ADC	Acute myeloid leukemia	[312]	2017; 2000 #	2018
Immunologic	Spesolimab	SPEVIGO^®^	IL-36 receptor	Humanized IgG1	Generalized pustular psoriasis	[313]	2022	2022
Immunologic	Anifrolumab	Saphnelo	IFNAR1	Human IgG1	Systemic lupus erythematosus	[314]	2021	2022
Immunologic	Bimekizumab	Bimzelx	IL-17A,F	Humanized IgG1	Psoriasis	[315]	2nd cycle review	2021
Immunologic	Tralokinumab	Adtralza	IL-13	Human IgG4	Atopic dermatitis	[316]	2021	2021
Immunologic	Risankizumab	Skyrizi	IL-23p19	Humanized IgG1	Plaque psoriasis	[317]	2019	2019
Immunologic	Emapalumab	Gamifant	IFN-ɣ (gamma)	Human IgG1	Primary hemophagocytic lymphohistiocytosis	[318]	2018	NA
Immunologic	Lanadelumab	Takhzyro	Plasma kallikrein	Human IgG1	Hereditary angioedema attacks	[319]	2018	2018
Immunologic	Tildrakizumab	Ilumya	IL-23p19	Humanized IgG1	Plaque psoriasis	[320]	2018	2018
Immunologic	Ibalizumab	Trogarzo	CD4	Humanized IgG4	HIV infection	[321]	2018	2019
Immunologic	Guselkumab	TREMFYA	IL-23 P19	Human IgG1	Plaque psoriasis	[322]	2017	2017
Immunologic	Sarilumab	Kevzara	IL-6R	Human IgG1	Rheumatoid arthritis	[323]	2017	2017
Immunologic	Dupilumab	Dupixent	IL-4Rα	Human IgG4	Atopic dermatitis	[324]	2017	2017
Immunologic	Brodalumab	Siliq, LUMICEF	IL-17R	Human IgG2	Plaque psoriasis	[325]	2017	2017
Immunologic	Ixekizumab	Taltz	IL-17a	Humanized IgG4	Psoriasis	[326]	2016	2016
Immunologic	Secukinumab	Cosentyx	IL-17a	Human IgG1	Psoriasis	[327]	2015	2015
Immunologic	Siltuximab	Sylvant	IL-6	Chimeric IgG1	Castleman disease	[328]	2014	2014
Immunologic	Belimumab	Benlysta	BLyS	Human IgG1	Systemic lupus erythematosus	[329]	2011	2011
Immunologic	Tocilizumab	RoActemra, Actemra	IL-6R	Humanized IgG1	Rheumatoid arthritis	[330]	2010	2009
Immunologic	Canakinumab	Ilaris	IL-1β	Human IgG1	Muckle-Wells syndrome	[331]	2009	2009
Immunologic	Golimumab	Simponi	TNF	Human IgG1	Rheumatoid and psoriatic arthritis, ankylosing spondylitis	[332], [333]	2009	2009
Immunologic	Ustekinumab	Stelara	IL-12/23	Human IgG1	Psoriasis	[334]	2009	2009
Immunologic	Efalizumab	Raptiva	CD11a	Humanized IgG1	Psoriasis	[335]	2003 #	2004 #
Immunologic	Adalimumab	Humira	TNF	Human IgG1	Rheumatoid arthritis	[336]	2002	2003
Immunologic	Muromonab-CD3	Orthoclone Okt3	CD3	Murine IgG2a	Reversal of kidney transplant rejection	[3]	1986 #	1986 *
Immunologic	Basiliximab	Simulect	IL-2R	Chimeric IgG1	Prevention of kidney transplant rejection	[337]	1998	1998
Immunologic	Daclizumab	Zenapax; Zinbryta	IL-2R	Humanized IgG1	Prevention of kidney transplant rejection; multiple sclerosis	[338]	2016; 1997 #	2016; 1999 #
Immunologic	Lebrikizumab	(Pending)	IL-13	humanized IgG4	Atopic dermatitis	[339]	NA	Review
Oncologic	Enfortumab vedotin	Padcev	Nectin-4	Human IgG1 ADC	Urothelial cancer	[340]	2019	2022
Oncologic	Catumaxomab	Removab	EPCAM/CD3	Rat/mouse bispecific mAb	Malignant ascites	[341]	NA	2009 #
Oncologic	Ipilimumab	Yervoy	CTLA-4	Human IgG1	Metastatic melanoma	[342]	2011	2011
Oncologic	Tebentafusp	KIMMTRAK	gp100, CD3	Bispecific immunoconjugate (TCR-scFv)	Metastatic uveal melanoma	[343]	2022	2022
Oncologic	Relatlimab	Opdualag (relatlimab + nivolumab combo)	LAG-3	Human IgG4	Melanoma	[344]	2022	2022
Oncologic	Tremelimumab	Imjudo	CTLA-4	Human IgG2A	Antineoplastic; liver cancer	[345]	2022	2023
Oncologic	Retifanlimab	Zynyz	PD-1	Humanized IgG4	Merkel-cell carcinoma	[346]	2023	In review
Oncologic	Penpulimab	(Pending)	PD-1	Humanized IgG1	Metastatic nasopharyngeal carcinoma	[347]	Review status unknown	NA
Oncologic	Toripalimab	Tuoyi	PD-1	Humanized IgG4	Nasopharyneal carcinoma, esophageal squamous-cell carcinoma	[348], [349]	Decision delayed	Review
Oncologic	Tislelizumab	(Pending)	PD-1	Humanized IgG4	Esophageal squamous-cell carcinoma	[350]	2nd cycle review	Review
Oncologic	Cosibelimab	(Pending)	PD-L1	Human IgG1	Squamous-cell carcinoma	[351]	Review	NA
Oncologic	Naxitamab	DANYELZA	GD2	Humanized IgG1	High-risk neuroblastoma and refractory osteomedullary disease	[271]	2020	NA
Oncologic	Trastuzumab duocarmazine	(Pending)	HER2	Humanized IgG1 ADC	Breast cancer	[352]	2nd cycle review	Review
Oncologic	Mirvetuximab soravtansine	ELAHERE	Folate receptor α	Humanized IgG1 ADC	Ovarian cancer	[353]	2022	NA
Oncologic	Tisotumab vedotin	TIVDAK	Tissue factor	Human IgG1 ADC	Cervical cancer	[354]	2021	NA
Oncologic	Amivantamab	Rybrevant	EGFR, cMET	Human bispecific IgG1	NSCLC w/EGFR exon 20 insertion mutations	[355]	2021	2021
Oncologic	Dostarlimab	Jemperli	PD-1	Humanized IgG4	Endometrial cancer	[356]	2021	2021
Oncologic	Margetuximab	MARGENZA	HER2	Chimeric IgG1	HER2+ breast cancer	[357]	2020	NA
Oncologic	Sacituzumab govitecan	Trodelvy	TROP-2	Humanized IgG1 ADC	Triple-neg. breast cancer	[358]	2020	2021
Oncologic	[fam]-trastuzumab deruxtecan	Enhertu	HER2	Humanized IgG1 ADC	HER2+ breast cancer	[359]	2029	2021
Oncologic	Cemiplimab	Libtayo	PD-1	Human IgG4	Cutaneous squamous-cell carcinoma	[360]	2018	2019
Oncologic	Durvalumab	IMFINZI	PD-L1	Human IgG1	Bladder cancer	[361]	2017	2018
Oncologic	Avelumab	Bavencio	PD-L1	Human IgG1	Merkel-cell carcinoma	[362]	2017	2017
Oncologic	Atezolizumab	Tecentriq	PD-L1	Humanized IgG1	Bladder cancer	[363]	2016	2017
Oncologic	Olaratumab	Lartruvo	PDGRFα	Human IgG1	Soft tissue sarcoma	[364]	2016 #	2016 #
Oncologic	Dinutuximab	Qarziba; Unituxin	GD2	Chimeric IgG1	Neuroblastoma	[365]	2015	2017; 2015 #
Oncologic	Pembrolizumab	Keytruda	PD1	Humanized IgG4	Melanoma	[366]	2014	2015
Oncologic	Ramucirumab	Cyramza	VEGFR2	Human IgG1	Gastric cancer	[367]	2014	2014
Oncologic	Ado-trastuzumab emtansine	Kadcyla	HER2	Humanized IgG1, ADC	Breast cancer	[368]	2013	2013
Oncologic	Pertuzumab	Perjeta	HER2	Humanized IgG1	Breast Cancer	[369]	2012	2013
Oncologic	Panitumumab	Vectibix	EGFR	Human IgG2	Colorectal cancer	[370]	2006	2007
Oncologic	Bevacizumab	Avastin	VEGF	Humanized IgG1	Colorectal cancer	[371]	2004	2005
Oncologic	Cetuximab	Erbitux	EGFR	Chimeric IgG1	Colorectal cancer	[372]	2004	2004
Oncologic	Trastuzumab	Herceptin	HER2	Humanized IgG1	Breast cancer	[97]	1998	2000
Cardiovascular	Evinacumab	Evkeeza	Angiopoietin-like 3	Human IgG4	Homozygous familial hypercholesterolemia	[373]	2021	2021
Cardiovascular	Alirocumab	Praluent	PCSK9	Human IgG1	High cholesterol	[124]	2015	2015
Cardiovascular	Evolocumab	Repatha	PCSK9	Human IgG2	High cholesterol	[374]	2015	2015
Cardiovascular	Abciximab	Reopro	GPIIb/IIIa	Chimeric IgG1 Fab	Prevention of blood clots in angioplasty	[375]	1994	1995 *
Endocrine	Teprotumumab	Tepezza	IGF-1R	Human IgG1	Thyroid eye disease	[376]	2020	NA
Gastrointestinal	Pozelimab	(Pending)	Complement 5	Human IgG4	CHAPLE disease	[377]	Review	NA
Gastrointestinal	Mirikizumab	(Pending)	IL-23p19	Humanized IgG4	Ulcerative Colitis	[378]	Review	Review
Gastrointestinal	Vedolizumab	Entyvio	A4β7 integrin	Humanized IgG1	Ulcerative Colitis, Crohn’s Disease	[379]	2014	2014
Gastrointestinal	Certolizumab pegol	Cimzia	NF	Humanized Fab, pegylated	Crohn’s Disease	[170]	2008	2009
Gastrointestinal	Infliximab	Remicade	TNF	Chimeric IgG1	Crohn’s Disease	[380]	1998	1999
Gastrointestinal	Camrelizumab	(Pending)	PD-1	Human IgG4	Hepatocellular carcinoma	[381]	Review	NA
Gastrointestinal	Edrecolomab	Panorex	EpCAM	Murine IgG2a	Colorectal cancer	[382]	NA	1995 *#
Gastrointestinal	Bezlotoxumab	Zinplava	*Clostridium difficile* enterotoxin B	Human IgG1	Prevention of *Clostridium difficile* infection recurrence	[383]	2016	2017
Infectious disease	Ansuvimab	Ebanga	Ebola virus	Human IgG1	Ebola infection	[208]	2020	NA
Infectious disease	Atoltivimab, Maftivimab, and Odesivimab-ebgn	Inmazeb	Ebola virus	mixture of 3 human IgG1	Ebola virus infection	[209]	2020	NA
Infectious disease	Raxibacumab	(Pending)	B. anthrasis PA	Human IgG1	Anthrax infection	[384]	2012	NA
Infectious disease	Nebacumab	Centoxin	Endotoxin	Human IgM	Gram-negative sepsis	[385]	NA	1991 *#
Neurologic	Donanemab	(Pending)	Amyloid β	Humanized IgG1	Alzheimer’s disease	[386]	2nd cycle review	NA
Neurologic	Lecanemab	Leqembi	Amyloid β protofibrils	Humanized IgG1	Alzheimer’s disease	[387]	2023	Review
Neurologic	Omburtamab	(Pending)	B7-H3	Murine IgG1	CNS/leptomeningeal metastasis from neuroblastoma	[388]	2nd cycle review	Neg. opinion
Neurologic	Faricimab	Vabysmo	VEGF-A, Ang-2	Human/humanized IgG1 κ (kappa)/lambda, with domain crossover	wAMD, DME	[389]	2022	2022
Neurologic	Aducanumab	Aduhelm	Amyloid β	Human IgG1	Alzheimer’s disease	[254]	2021	MAA withdrawn
Neurologic	Satralizumab	Enspryng	IL-6R	Humanized IgG2	Neuromyelitis optica and neuromyelitis optica spectrum disorders	[390]	2020	2021
Neurologic	Inebilizumab	Uplizna	CD19	Humanized IgG1	Neuromyelitis optica and neuromyelitis optica spectrum disorders	[391]	2020	2022
Neurologic	Eptinezumab	Vyepti	CGRP	Humanized IgG1	Migraine prevention	[392]	2020	2022
Neurologic	Brolucizumab	BEOVU	VEGF-A	Humanized scFv	Macular degeneration	[393]	2019	2020
Neurologic	Fremanezumab	Ajovy	CGRP	Human IgG2	Migraine prevention	[266]	2018	2019
Neurologic	Galcanezumab	Emgality	CGRP	Human IgG4	Migraine prevention	[394]	2018	2018
Neurologic	Erenumab	Aimovig	CGRP receptor	Human IgG2	Migraine prevention	[395]	2018	2018
Neurologic	Ranibizumab	Lucentis	VEGF	Humanized IgG1 Fab	Macular degeneration	[396]	2006	2007
Neurologic	Natalizumab	Tysabri	a4 integrin	Humanized IgG4	Multiple sclerosis	[397]	2004	2006
Pulmonary	Serplulimab	(Pending)	PD-1	Human IgG4	Small-cell lung cancer	[398]	NA	Review
Pulmonary	Sugemalimab	(Pending)	PD-1	Human IgG4	Non-small-cell cancer	[399]	NA	Review
Pulmonary	Sintilimab	(Pending)	PD-1	Human IgG4	Non-small-cell lung cancer	[400]	2nd Cycle Review	NA
Pulmonary	Nirsevimab	Beyfortus	RSV	Human IgG1	RSV Infection	[401]	Review	2022
Pulmonary	Tixagevimab, cilgavimab	Evusheld	SARS-CoV-2	Human IgG1	COVID-19	[402]	EUA	2022
Pulmonary	Sotrovimab	Xevudy	SARS-CoV-2	Human IgG1	COVID-19	[403]	NA	2021
Pulmonary	Regdanvimab	Regkirona	SARS-CoV-2	Human IgG1	COVID-19	[404]	NA	2021
Pulmonary	Casirivimab + imdevimab	REGEN-COV, Ronapreve	SARS-CoV-2	Human IgG1	COVID-19	[405]	Review	2021
Pulmonary	Tezepelumab	Tezspire	Thymic stromal lymphopoeitin	Human IgG2	Severe Asthma	[406]	2021	2022
Pulmonary	Benralizumab	Fasenra	IL-5R α	Humanized IgG1	Asthma	[407]	2017	2018
Pulmonary	Reslizumab	Cingaero, Cingair	IL-5	Humanized IgG4	Asthma	[408]	2016	2016
Pulmonary	Obiltoxaximab	Anthim	Protective antigen of B.anthracis exotoxin	Chimeric IgG1	Preventionof inhalation anthrax	[409]	2016	2020
Pulmonary	Necitumumab	Portazza	EGFR	Human IgG1	Non-small-cell lung cancer	[410]	2015	2015
Pulmonary	Mepolizumab	Nucala	IL-5	Humanized IgG1	Severe eosinophilic asthma	[411]	2015	2015
Pulmonary	Nivolumab	Opdivo	PD1	Human IgG4	Non-small-cell lung cancer, melanoma	[90,412]	2014	2015
Pulmonary	Omalizumab	Xolair	IgE	Humanized IgG1	Asthma	[106]	2003	2005
Pulmonary	Palivizumab	Synagis	RSV	Humanized IgG1	Prevention of RSV infection	[205]	1998	1999
Muscoloskeletal	Romosozumab	Evenity	Sclerostin	Humanized IgG2	Osteoporosis in postmenopausal women at risk of fracture	[273]	2019	2019
Musculoskeletal	Burosumab	Crysvita	FGF23	Human IgG1	X-linked hypophosphatemia	[276]	2018	2018
Musculoskeletal	Denosumab	Prolia	RANK-L	Human IgG2	Bone Loss	[413]	2010	2010
Musculoskeletal	Rozanolixizumab	RYSTIGGO^®^	FcRn	Humanized IgG4	Generalized myasthenia gravis	[414]	2023	Review

## Data Availability

Not applicable.
